# Structural and functional characterization of IgG- and non-IgG-based T-cell-engaging bispecific antibodies

**DOI:** 10.3389/fimmu.2024.1376096

**Published:** 2024-05-28

**Authors:** Nishant Mohan, Safiat Ayinde, Hanjing Peng, Shraboni Dutta, Yi Shen, Vincent M. Falkowski, Thomas G. Biel, Tongzhong Ju, Wen Jin Wu

**Affiliations:** ^1^ Office of Pharmaceutical Quality Research, Office of Pharmaceutical Quality, Center for Drug Evaluation and Research, U.S. Food and Drug Administration, Silver Spring, MD, United States; ^2^ Office of Pharmaceutical Manufacturing Assessment, Office of Pharmaceutical Quality, Center for Drug Evaluation and Research, U.S. Food and Drug Administration, Silver Spring, MD, United States

**Keywords:** bispecific antibody, bispecific T-cell engager, epidermal growth factor receptor (EGFR), cluster of differentiation 3 (CD3), dual variable domain immunoglobulins (DVD-Ig)

## Abstract

Bispecific T-cell-engaging antibodies are a growing class of therapeutics with numerous molecules being tested in clinical trials and, currently, seven of them have received market approval. They are structurally complex and function as adaptors to redirect the cytotoxicity of T cells to kill tumor cells. T-cell-engaging bispecific antibodies can be generally divided into two categories: IgG/IgG-like and non-IgG-like formats. Different formats may have different intrinsic potencies and physiochemical properties, and comprehensive studies are needed to gain a better understanding of how the differences in formats impact on structural and functional characteristics. In this study, we designed and generated bispecific T-cell-engaging antibodies with IgG-like (DVD-Ig) and non-IgG (BiTE) formats. Both target the same pair of antigens (EGFR and CD3) to minimize the possible influence of targets on functional characterization. We performed a side-by-side comparison to assess differences in the physiochemical and biological properties of these two bispecific T-cell-engaging antibodies using a variety of breast and ovarian cancer cell-based functional assays to delineate the structural–functional relationships and anti-tumor activities/potency. We found that the Fc portion of T-cell-engaging bispecific antibodies can significantly impact antigen binding activity, potency, and stability in addition to eliciting different mechanisms of action that contribute the killing of cancer cells.

## 1 Introduction

T-cell-engaging bispecific antibodies (BsAbs) have emerged as an important class of targeted immunotherapy and revolutionized the clinical management of malignant tumors. CD3-based T-cell-engaging bispecific antibodies are potent molecules to elicit T-cell-mediated anti-tumor response because of their ability to simultaneously bind to tumor-associated antigen (TAA) with one arm and CD3 expressed on T cell with other arm, thereby, redirecting patient’s own cytotoxic T cells to eliminate cancer cells (1, 2). T cells express T-cell receptor (TCR) alpha and beta subunits, which are noncovalently associated with six CD3 subunits (3). Naturally, when antigen-specific peptide/major histocompatibility complex (MHC) expressed on tumor cells engages with TCR-CD3 complex on T cells, T cells activate TCR-mediated signaling cascade. However, T-cell engagers targeting CD3 offer unique advantage of bypassing the need for TCR/MHC complex interaction. T-cell engagers facilitate the formation of an immunological synapse on the surface of tumor cells by linking TAA with the CD3ϵ unit of the TCR complex. Since these molecules target CD3_ε_ subunit on TCR complex, it can activate the entire repertoire of T cells irrespective of TCR specificity and trigger T-cell responses without costimulatory signals such as CD28 and interleukin 2 (2, 4, 5).

Broadly, T-cell-engaging BsAb constructs can be generally divided into two categories: IgG-like format containing Fc domains and non-IgG-like format without Fc domains. The advantage of having Fc domains can be attributed to improving the stability of the molecule, simplifying the manufacturing process, and extending the half-life of the BsAbs (6–8); however, the tissue permeability of IgG-based molecules is generally lower than non-IgG-like format due to the higher molecular weight (2). Fc domains in BsAb molecules have the potential to exert Fc-related effector functions such as antibody dependent cell-mediated cytotoxicity (ADCC) and complement dependent cytotoxicity (CDC) (9), although, these functions can be eliminated by genetically silencing Fc domain functions while still retaining the solubility, stability, extended half-life, and purification advantages. One of the commonly used IgG or IgG-like BsAb formats for clinical development is the dual variable domain (VD) immunoglobulins (DVD-Ig), which is generated by connecting an additional VD via a linker sequence at the N termini of the VH and VL of an existing monoclonal antibody (mAb) to yield a tetravalent IgG-like molecule (10, 11). On the other hand, scFv-based non-IgG BsAb are smaller in molecular size due to the absence of Fc domain and generated by fusing two single-chain variable fragments (scFvs) from antibodies against TAA and CD3 connected with a short linker. Three major non-IgG-based BsAb formats have been developed, including bispecific T-cell engager (BiTE), dual-affinity retargeting antibody (DART), and tandem diabody (TandAb) (12, 13). Among them, the BiTE non-IgG format consists of anti-CD3 scFv and TAA scFv that are connected by a peptide linker. Currently, seven CD3-based T-cell-engaging antibodies (elranatamab, talquetamab, epcoritamab, glofitamab, teclistamab, mosunetuzumab, and blinatumomab) have received approval for the U.S. market (accessed on November 2023 https://www.antibodysociety.org/resources/approved-antibodies/). Blinatumomab is the first BiTE non-IgG format approved for treatment of B-cell malignancy, whereas other recently approved T-cell engagers are based on the IgG-like format. BiTE-based format offers numerous advantages due to its a small and flexible structure, which can easily diffuse and can quickly move from the site of administration to the site of lesions, recruit cytotoxic T cells to cancer cells with high affinity.

Epidermal growth factor receptor (EGFR) is frequently overexpressed in both ovarian cancers (OCs) and triple-negative breast cancers (TNBCs); however, the anti-EGFR mAb cetuximab has not been approved for treatment of OC or TNBC patients, despite its potent anti-tumor activity in pre-clinical models (14–16). Immune checkpoint inhibitors have shown clinical effectiveness for many tumor types, including gynecological malignancies. However, these therapeutic successes have not been demonstrated in OC clinical studies (17).

Although we have gained some aspects of understanding in terms of advantages and disadvantages for IgG and non-IgG formats of bispecific antibodies, it is largely obtained from preclinical studies. There is still a lack of knowledge from studies that performed a side-by-side comparison of these two different formats of bispecific antibodies that target the same pair of antigens from product quality perspective. In this investigation, we designed and generated T-cell engager bispecific antibodies with two molecular formats, DVD-Ig and BiTE, targeting EGFR expressed on the cancer cells and CD3 expressed on T cell (hereby referred as anti-EGFR/CD3 BsAbs). We performed a side-by-side comparison to assess differences in physiochemical and biological properties of these two T-cell-engaging bispecific antibodies using a variety of biochemical and functional assays to delineate the structural/functional relationships and anti-tumor potency and activities.

## 2 Materials and methods

### 2.1 Cell lines and reagents

The OC cell lines (SKOV3, CaOV3, PA1, NIH-OVCAR3, and SW126) and the breast cancer cell line (MDA-MB-231) were obtained from American Type Culture Collection (Manassas, VA, USA) and propagated at 37°C under 5% CO_2_ in culture media conditions as described in manufacture’s protocol. OC cells (A2780 and A2780cis) were purchased from Sigma-Aldrich (St. Louis, MO). The therapeutic monoclonal antibodies, cetuximab (Erbitux) and blinatumomab (Blincyto), were purchased from the Food and Drug Administration (FDA)–designated pharmacy (WEP Clinical, Morrisville, MC). The EGF, recombinant CD3, and recombinant EGFR were obtained from RayBiotech (Peachtree Corners, GA, USA). The control bispecific antibodies anti-βGal-hCD3 (cat# bimab-bgalhcd3) and anti-hCD19-βGal (cat# bimab-hcd19bgal) were obtained from InvivoGen (San Diego, CA).

### 2.2 Generation of bispecific constructs and purification

The non-IgG-based BiTE format of anti-EGFR/CD3 BsAb was generated by connecting two single-chain variable (scFv) regions from anti-EGFR and anti-CD3 antibodies via a glycine linker. The IgG-based DVD-Ig format was generated by connecting an additional VD from anti-CD3 via a linker sequence at the N termini of the VH and VL of cetuximab to yield a tetravalent IgG-like molecule. The amino acid sequences of the anti-EGFR scFv regions were obtained from the anti-EGFR mAb (cetuximab) and the anti-CD3 scFv regions were obtained from the anti-CD3/CD19 BsAb (blinatumomab), which are publicly available on International Immunogenetics Information System (http://www.imgt.org), and bispecific antibodies were generated and produced using standard DNA recombinant technologies (18, 19). A single plasmid encoding BiTE format and two plasmids encoding heavy and light chains of DVD-Ig format were constructed in pcDNA3.1 (+) vector and then transiently transfected into CHO cells to produce BsAb proteins (Genscript Piscataway, NJ, USA). The BiTE protein was generated with hexa-histidine (6 HIS) affinity tag, purified using one-step HisTrap FF crude columns, and eluted using gradient of imidazole. The DVD-Ig protein was purified using MabSelect SuRE protein A affinity columns. The purified proteins were formulated in phosphate-buffered saline (PBS) and stored at −80°C until further use. Production yield was approximately 0.06–0.14 mg/ml for BiTE and 0.7–0.9 mg/ml for DVD-Ig from 100 ml of culture supernatant.

### 2.3 HPLC assay

High-performance liquid chromatography size exclusion chromatography is an analytical method technique used to characterize and monitor protein-based purity (20). HPLC experiments were performed on Agilent 1260 Infinity LC system using AdvancedBio SEC 300 Å 4.6 mm × 300 mm, 2.7 µm columns (Agilent technologies, Santa Clara, CA). The mobile phase consisted of 150 mM sodium phosphate buffer at pH 7.0. Samples were eluted for 30 min at the flow rate of 0.35 µl/min and detected at 280 nm absorbance. Chromatograms were subjected to area under the curve analyses using OpenLAB CDS software version 2.7 from Agilent.

### 2.4 CE-SDS method

Capillary electrophoresis sodium dodecyl sulfate (CE-SDS) PLUS on the Maurice (ProteinSimple, San Jose, CA) was performed in non-reducing conditions per the manufacture’s recommendations. CE-SDS PLUS reagents, cartridges, and materials were purchased from ProteinSimple. The BiTE and DVD-Ig samples and an IgG standard were subjected to CE-SDS PLUS experiments as per manufacturer’s instructions described in the Maurice CE-SDS application guide. Briefly, samples were diluted to 0.25 μg/μL in 1× Maurice CE-SDS PLUS sample buffer, and 2 μL of 25× internal standard was added per 50 μl of sample. The lyophilized IgG standard from ProteinSimple was reconstituted in 50 μl of 1× Maurice CE-SDS PLUS Sample Buffer and 2 μL of 25× internal standard was added. For non-reducing conditions, 2.5 μL of 250 mM iodoacetamide was added to the samples and IgG standard, mixed briefly by vortex, and then heated at 70°C for 10 min. After cooling the samples on ice for 4 min, 50 μl of the samples and a non-reduced IgG standard were transferred to a 96-well plate that was placed inside the Maurice system to analyze the samples. The BiTE and DVD-Ig samples and the IgG standard were injected onto a Maurice CE-SDS PLUS Cartridge at 4.6 kV for 20 s, and the samples were separated at 5.75 kV for 60 min, and the non-reduced IgG standard was separated at 5.75 kV for 35 min. The results of the ran CE-SDS PLUS samples was obtained using Compass for iCE Version 4.0.0 (ProteinSimple, San Jose, CA).

### 2.5 Flow cytometry binding experiments

The cell surface binding of anti-EGFR/CD3 BsAbs with EGFR expressed on tumor cells and CD3 expressed on T cells was detected using flow cytometry as described previously (21–23). Briefly, tumor cells (CaOV3, PA1, and MDA MB 231 cells) and T cells were washed with PBS and then stained with 1 µg of anti-EGFR/CD3 DVD-Ig, anti-EGFR/CD3 BiTE, cetuximab, blinatumomab and anti-βGal-hCD3 (control) in FACS buffer [1% fetal bovine serum (FBS) in PBS] for 1h. Cetuximab serves as positive and anti-βGal-hCD3 serve as negative controls for tumor cells. Blinatumomab and anti-βGal-hCD3 serve as positive and anti-βGal-hCD19 serves as negative controls for T cells. After washing with PBS, samples were incubated with FITC-conjugated secondary antibodies for 30 min. Samples were washed again, resuspended in FACS buffer, and analyzed with LSR x-20 flow cytometer to determine the mean fluorescence intensity.

### 2.6 Immunoprecipitation binding experiments

The whole cell lysates (WCLs) from tumors cells (CaOV3, MDA-MB-231, and PA1) or T cells were incubated with 1 μg anti-EGFR/CD3 BiTE, anti-EGFR/CD3 DVD-Ig, anti-βGal-hCD19 (control for BiTE) cetuximab (positive control for DVD-Ig), and human IgG (negative control for DVD-Ig) overnight. Next day, 40 µl of protein L agarose beads were added to BiTE samples and 40 µl protein A agarose beads were added to cetuximab DVD-Ig and human IgG samples. Antibody-bound beads were washed three times with lysis buffer and then eluted with 2× lamellae sample buffer (Bio-Rad, Hercules, CA). Samples were heated for 5 min, centrifuged, and then loaded onto sodium dodecyl sulfate–polyacrylamide gel electrophoresis (SDS-PAGE). Tumor cell samples were probed with anti-EGFR antibody and T-cell samples were probed with anti-CD3 antibody followed by horseradish peroxidase–conjugated rabbit secondary antibody.

### 2.7 T-cell activation assay

T-cell activation assay is a bioluminescent cell-based assay, which is designed to reflect mechanism of action of anti-CD3-based antibodies using luminescence signal. In this assay, the Jurkat T cells were engineered to express a luciferase reporter gene driven by the NFAT-response element (NFAT-RE), which also expresses endogenous TCR, CD3, and CD28. When the TCR/CD3 effector cells (NFAT) are engaged with anti-TCR/CD3 antibody, the receptor-mediated signaling induces luminescence which can be detected and quantified using Bio-Glo™ Luciferase Assay System. T-cell activation assay was performed using 96-well format according to manufacturer’s instructions (Promega, cat# J1621). Briefly, approximately 20,000 tumor cells (CaOV3, PA1, MDA-MB-231, and MCF-7 cells) were seeded in white flat bottom 96-well plate using assay buffer (10% FBS in RPMI) and allowed to adhere overnight. Next day, serially diluted anti-EGFR/CD3 BsAbs protein and TCR/CD3 effector Cells (NFAT) were added into assay plate and incubated for 24h in a humid 37°C, 5% CO_2_ incubator. After the completion of incubation period, assay plates were removed from incubator and allow to equilibrate at room temperature. Bio-Glo™ Reagent was added to all the wells and luminescence signals were detected by using a GloMax plate reader (cat# GM3000, Promega, Madison, WI). The luminescence data was plotted as RLU versus Log_10_ concentration of antibodies.

### 2.8 T-cell-dependent cytotoxicity assay

T-cell-dependent cytotoxicity assays were performed in 96-well plate system. Briefly, target cancer cells (CaOV3, PA1, and MDA-MB-231) were cultured overnight in media containing 10% FBS in a 96-well plate. Next day, anti-EGFR/CD3 BsAbs protein samples (50 nM concentration) and TCR/CD3 effector T cells at an effector to target ratio of 3:1 was added to the 96-well assay plate containing target cancer cells. Assay plates were incubated for 24h or 48h. After the incubation period, Promega CellTiterGlo reagent was added, and luminescence signals was read using Promega GloMax plate reader.

### 2.9 Granzyme B, perforin measurement, and cytokine array

Quantitative measurement of Granzyme B was performed using Human Granzyme B Quantikine ELISA Kit (cat# DGZB00, R and D Systems, Minneapolis, MN) and perforin was measured using Perforin (PRF1) Human ELISA Kit (cat# ab46068, Abcam, Waltham, MA). Cytokine detection was performed by subjecting the samples to Proteome Profiler Human XL Cytokine Array Kit (cat# ARY022B, R, and D Systems), which is a membrane-based sandwich immunoassay. For these experiments, co-culture of cancer cells (CaOV3 and MDA-MB-231) and T-cells were treated with BsAb proteins for 48h. After the incubation, co-culture media was harvested, centrifuged and then supernatant was collected. Co-culture supernatants from different treatment conditions were subjected to Granzyme B and Perforin ELISA kits and cytokine array kit as described in their respective manufacturer’s protocols.

### 2.10 SDS-PAGE and Western blotting

BiTE and DVD-Ig BsAb protein samples were analyzed by SDS-PAGE after subjecting the samples under reducing and non-reducing conditions. For non-reduced SDS-PAGE analysis, BsAb protein samples were prepared in Tris-HCL and SDS containing Laemmli sample buffer (cat# 1610737, Bio-Rad). For reducing conditions, 2-mercaptoethanol was added into sample buffer as a reducing agent prior to preparing the samples. After heating the BsAb samples for 5 min, protein bands were separated using 4%–15% gradient SDS-PAGE gel, stained with SimplyBlue Coomassie stain (cat# LC6065, Thermo Fisher Scientific, Waltham, MA) for 1h, washed with deionized water and then visualized with ChemiDoc MP gel imaging system (Bio-Rad). For Western blot analysis, whole-cell lysates were prepared after lysing cells in NP-40 lysis buffer and then mixed with in sample buffer containing Tris-HCL and SDS. Protein bands were separated using 4%–15% gradient SDS-PAGE gels under reducing conditions as described previously (21, 22). Gels were transferred to a 0.2-µm PVDF membrane using Trans-Blot Turbo Transfer system (Bio-Rad). After blocking in 5% non-fat milk, membranes were probed with primary antibodies overnight at 4°C and secondary antibodies for 1h at room temperature.

### 2.11 Statistical analysis

The statistical significance was determined using Microsoft Excel and GraphPad Prism software and data were compared by a two-tailed Student’s *t*-test. The data was presented in the form of mean and error bars represent standard deviation. The differences between two groups were considered statistically significant when *p* < 0.05 (*p < 0.05; **p < 0.01).

## 3 Results

### 3.1 Structure, purity, and binding characterization of anti-EGFR/CD3 BiTE and DVD-Ig BsAbs

**Figure 1:**
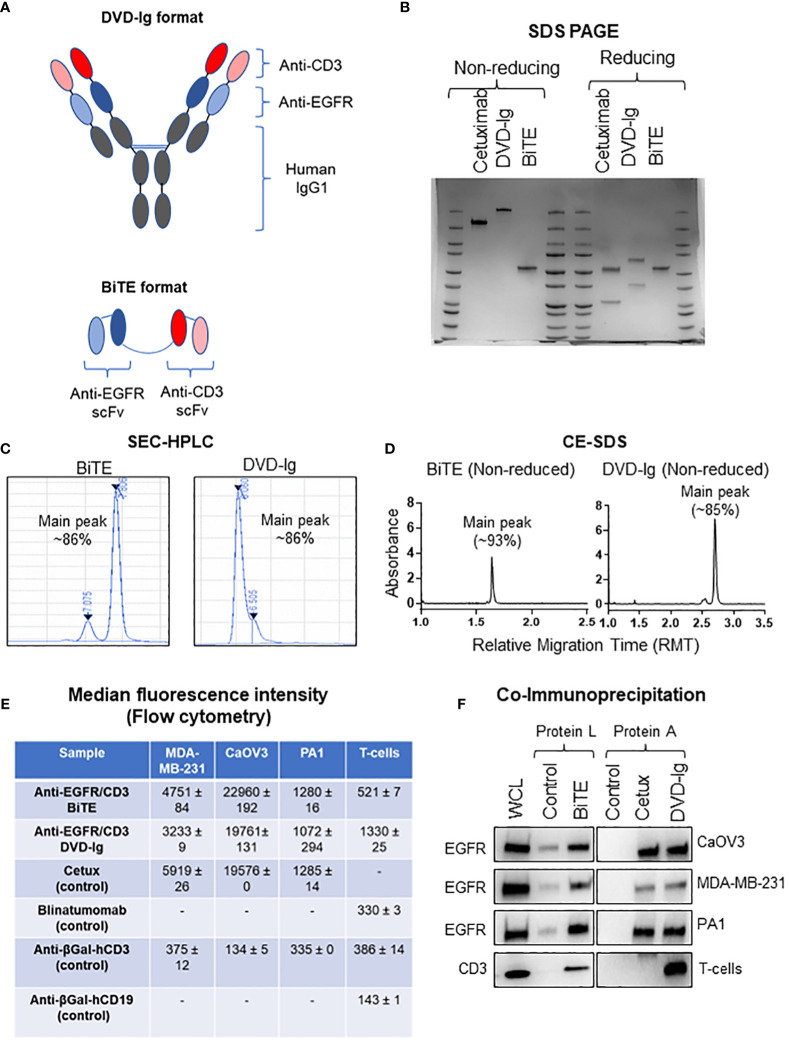
Physiochemical properties of anti-EGFR/CD3 DVD-Ig and BiTE BsAbs. **(A)** Schematic illustration of DVD-Ig and BiTE formats of BsAbs targeting EGFR and CD3. **(B)** SDS-PAGE analysis of anti-EGFR/CD3 DVD-Ig and BiTE BsAbs under non-reducing and reducing conditions. **(C)** Representative chromatograms of DVD-Ig and BiTE BsAb protein sample using the size-exclusion chromatography HPLC method. **(D)** Representative electropherogram of DVD-Ig and BiTE BsAb protein samples using the non-reduced capillary electrophoresis (CE)–SDS method. **(E)** Median fluorescence intensity (MFI) determined by flow cytometry after staining cells with anti-EGFR/CD3 DVD-Ig and BiTE BsAbs. “-”denotes experimental conditions not performed. Cetuximab as positive and anti-βGal-hCD3 as negative control for tumor cells. MFI values represent mean ± SD from duplicate experiments. Blinatumomab and anti-βGal-hCD3 as positive and anti-βGal-hCD19 as negative control for T cells. **(F)** Co-immunoprecipitation blots for anti-EGFR/CD3 DVD-Ig and BiTE BsAbs. Anti-βGal-hCD19 is used as negative control for BiTE. Cetuximab is used as positive control and human IgG as negative control for DVD-Ig. WCL represents whole cell lysates.

To perform the comparative characterization of non-IgG and IgG-like BsAb formats, we designed and produced anti-EGFR/CD3 BsAbs with either a non-IgG BiTE format or an IgG-like DVD-Ig format that target the same pair of antigens to minimize the possible influence of multiple targets during the functional characterization (**Figure 1A**). Using the anti-EGFR/CD3 BsAbs with different formats, we investigated the impact of the Fc domain on the physiochemical and biological properties of the BsAbs. The structural integrity was determined by SDS-PAGE analysis, in which an ~210 kDa major band for DVD-Ig and ~55 kDa for BITE was observed under non-reducing conditions (**Figure 1B**). Under SDS-PAGE reducing condition, an ~65 kDa heavy chain and ~36 kDa light chain for DVD-Ig format and a single band at ~55 kDa for BiTE was observed (**Figure 1B**). The purity of both formats was determined by SEC-HPLC and non-reduced CE-SDS methods. Purity as determined by the % area of main peak SEC-HPLC chromatogram was calculated as ~86% for both DVD-Ig and BiTE formats (**Figure 1C**). Purity by non-reduced CE-SDS method was determined as ~93% and ~85% for BiTE and DVD-Ig format, respectively (**Figure 1D**). These physicochemical characterization data suggest that both formats of anti-EGFR/CD3 BsAbs retain their respective structural integrity and have appropriate protein-based purity. Flow cytometry analysis has been commonly used to determine the binding specificity of bispecific T-cell-engaging antibodies to antigens (24). To determine the binding specificity of the BiTE- and DVD-Ig-formatted BsAbs to the EGFR and CD3 antigens, flow cytometry analysis and co-immunoprecipitation assay were performed using EGFR expressing tumor cells and CD3-expressing T cells (**Figures 1E, F**). The mean fluorescent intensity data obtained from flow cytometry staining indicated that both anti-EGFR/CD3 BsAb formats bound to EGFR expressed on all three tumor cells (MDA-MB-231, CaOV3, and PA1) as compared to anti-EGFR mAb (cetuximab), which served as positive control (**Figure 1E** and **Supplementary Figure S1**). The BiTE format showed a stronger binding toward EGFR expressed on three different cancer cells than DVD-Ig format. On the other hand, both formats also demonstrated binding activity toward CD3 expressed on T cells, but DVD-Ig format bound CD3 better than BiTE format (**Figure 1E**). Co-immunoprecipitation assay data, as shown in **Figure 1F**, further confirmed that both formats of BsAbs were capable of immunoprecipitating EGFR and CD3 specifically from EGFR-expressing cancer cells and T cells. Taken together, these data showed that dual binding functionalities of BiTE and DVD-Ig BsAbs remain intact.

### 3.2 T-cell-independent growth inhibition by anti-EGFR/CD3 BsAbs

**Figure 2:**
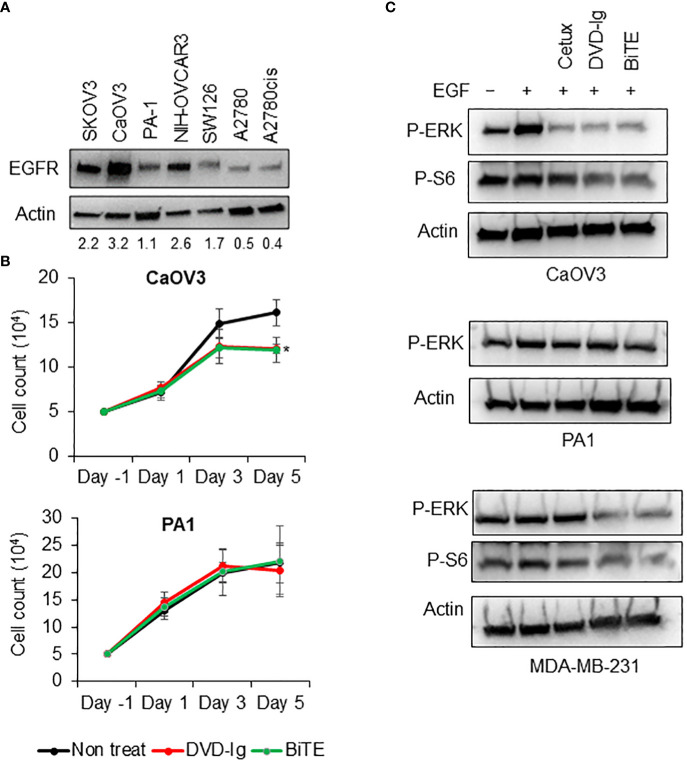
T-cell independent growth inhibitory activities of anti-EGFR/CD3 DVD-Ig and BiTE BsAbs **(A)** EGFR immunoblot using WCL from SKOV3, CaOV3, PA1, NIH-OVCAR3, SW126, A2780, and A2780cis. Actin was used as loading control. **(B)** Trypan blue cell proliferation assay using CaOV3 and PA1 cells after treatment of cells with anti-EGFR/CD3 BsAb at days 1, 3, and 5. Non-treated cells were used as control. This experiment was performed in biological triplicates, and data is presented as mean ± SD. The differences between two groups were considered statistically significant when *p* < 0.05 (*p < 0.05). **(C)** Anti-phosphorylated ERK and anti-phosphorylated S6 immunoblots using WCL from overnight serum starved CaOV3, PA1, and MDA-MB-231 cells subjected to anti-EGFR/CD3 BsAbs and cetuximab followed by EGF at 50 ng/mL concentration for 30 min.

EGFR overexpression is associated with poor prognosis and decreased therapeutic outcome for OC patients (15, 16). We first assessed the levels of EGFR expression in a panel of OC cells then, investigated the anti-tumor potencies of both anti-EGFR/CD3 BsAb formats independent of their T-cell-mediated activity. Western blotting data presented in **Figure 2A** indicated that CaOV3 cells had the highest expression of EGFR whereas PA1 expressed relatively lower EGFR on cell surface. Trypan blue cell proliferation assay showed that both anti-EGFR/CD3 BsAbs formats significantly inhibited cell proliferation of the high EGFR-expressing CaOV3 cells after exposure for 5 days, while the cell proliferation of the low-EGFR expressing PA1 cells subjected to both anti-EGFR/CD3 BsAbs was not inhibited (**Figure 2B**). The signaling mechanism of cell growth inhibition were further examined by assessing the phosphorylation levels of EGFR signaling downstream targets ERK1/2 and ribosomal protein S6 in CaOV3, PA1, and MDA-MB-231 cells. Treatment with EGF dramatically induced the ERK1/2 activation in CaOV3 cells but failed to activate in PA1 and MDA-MB-231 cells. Previous studied have indicated that PA1 cells contain an activated ras^N^ which corresponds to enhanced tumorigenicity of the cell line (25), whereas MDA-MB-231 possess BRAF, NF1, and oncogenic KRAS mutation exhibiting strong activation of RAS/MAP kinase signaling (26). Both formats of anti-EGFR/CD3 BsAbs significantly downregulated EGF-induced ERK1/2 activation in CaOV3 cells, which was also observed in cetuximab-treated cells (**Figure 2C**). ERK phosphorylation was not inhibited by cetuximab and BsAbs in PA1 cells (**Figure 2C**). Additionally, the phosphorylation of ribosomal protein S6, a downstream target of EGFR signaling and a critical regulator of cell size, cell proliferation, and protein synthesis (27, 28), was also downregulated in BsAb-treated CaOV3 cells. Both bispecific antibodies, but not cetuximab, inhibited EGF-induced phosphorylation of ERK and S6 kinase in MDA-MB-231 cell. Overall, T-cell-independent cell growth inhibition induced by both anti-EGFR/CD3 BsAb formats in high-EGFR expressing cells can be attributed to the EGFR-targeting arms of the BsAbs, which mimic the cetuximab-mediated signaling effects.

### 3.3 Target-dependent T-cell activation potency assay

**Figure 3:**
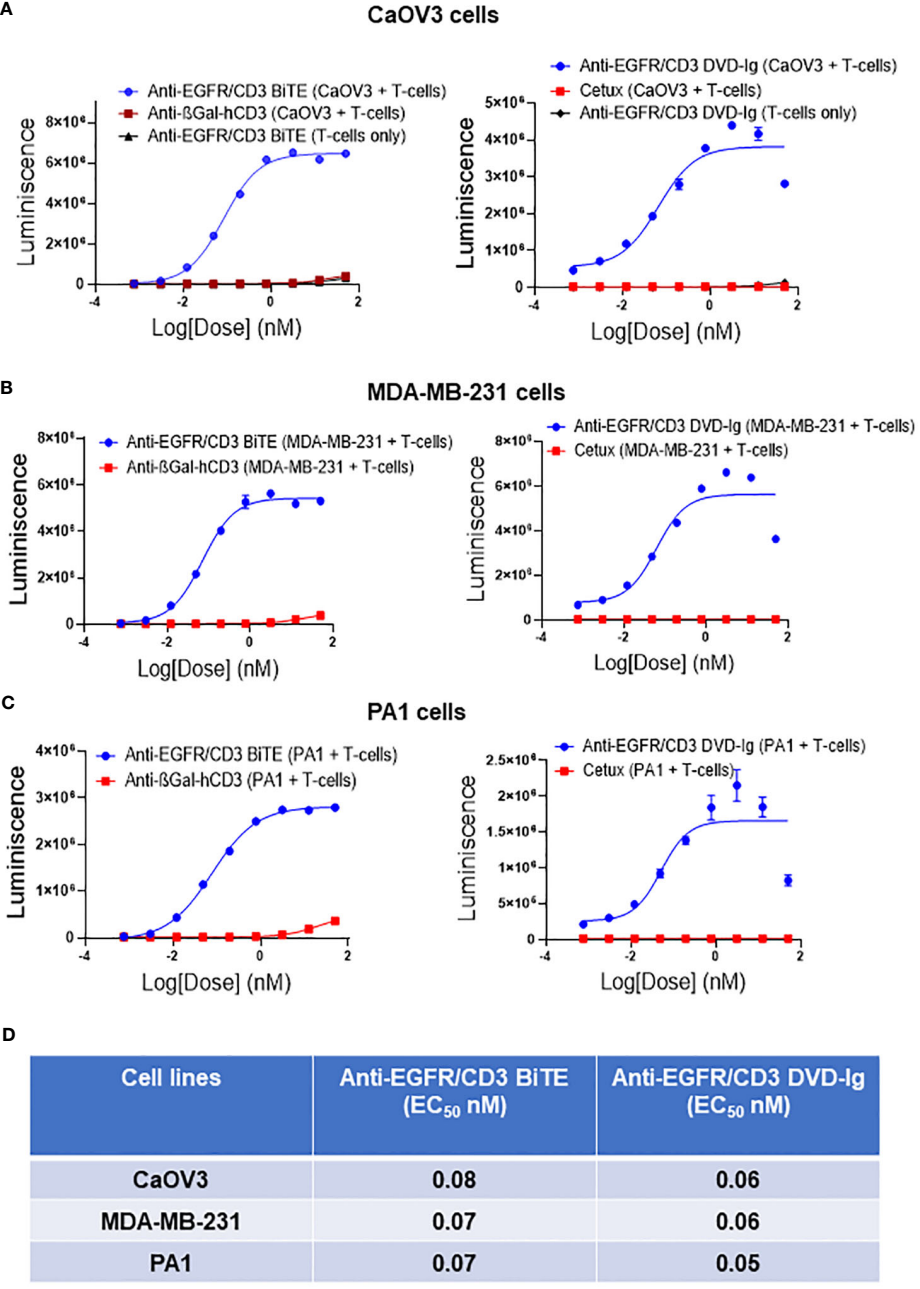
Target dependent T-cell activation by anti-EGFR/CD3 DVD-Ig and BiTE BsAbs Target cancer cells CaOV3 **(A)**, MDA-MB-231 **(B)** and PA1 **(C)** were co-cultured with TCR/CD3 effector T cells. Anti-βGal-hCD3 (red squares) is used as negative control for anti-EGFR/CD3 BiTE (blue circles). Cetuximab (red squares) is used as negative control for anti-EGFR/CD3 DVD-Ig (blue circles). In **(A)** anti-EGFR/CD3 BiTE or DVD-Ig (black triangles) were incubated with T cells only. This experiment was performed in biological triplicates, and data is presented as mean ± SD. **(D)** EC_50_ values of anti-EGFR/CD3 BiTE and DVD-Ig from T-cell activation assay in CaOV3, MDA-MB-231 and PA1 cells after 24h incubation.

The capability of anti-EGFR/CD3 BsAbs to redirect T cells toward EGFR-expressing tumor cells and induce TCR-mediated T-cell activation was determined by NFAT-luciferase bioluminescent cell-based bioassay. Solid tumor cells expressing EGFR (CaOV3, MDA-MB-231, and PA1) co-cultured with genetically engineered Jurkat T cells expressing a luciferase reporter were exposed to serial diluted anti-EGFR/CD3 BsAbs. Data shown in **Figures 3A–C** indicated that both formats of anti-EGFR/CD3 BsAbs effectively activated the reporter T cells in presence of EGFR-expressing tumor cell lines. To control the T-cell activation ability of anti-EGDR/CD3 BsAbs, a negative control anti-βGal-hCD3 was used in these assays, which has capability to bind to human CD3, but cannot activate reporter cells due to the absence of EGFR-binding capacity. In all three tumor cell lines tested, negative control anti-βGal-hCD3 did not display T-cell activation capabilities. For controlling the anti-EGFR/CD3 BsAb DVD-Ig format, cetuximab, a mAb against EGFR was used. Unlike DVD-Ig, cetuximab did not demonstrate the capability to activate the T cells. The T-cell activation ability was also measured in absence of tumor cell lines, which demonstrated that no T-cell activation was induced by BiTE or DVD-Ig format in absence of tumor antigen. Comparative analysis of EC_50_ values obtained from T-cell activation assay (**Figure 3D**) shows that both anti-EGFR/CD3 BsAb formats have comparable T-cell activation potency using these three tumor cell lines.

### 3.4 T-cell-dependent cytotoxicity assays

**Figure 4:**
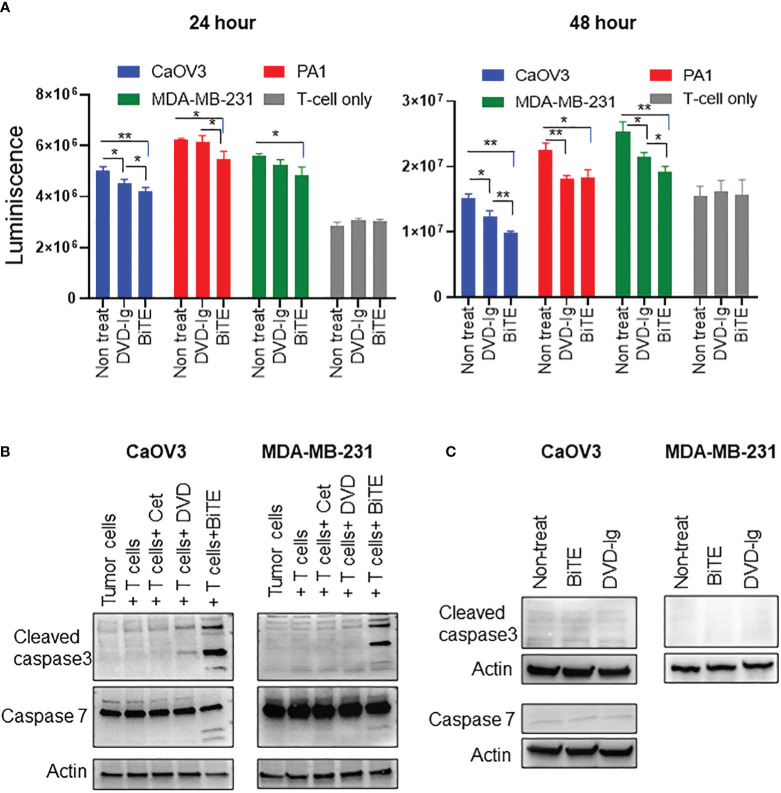
Target dependent T-cell-mediated cytotoxicity by anti-EGFR/CD3 DVD-Ig and BiTE BsAbs **(A)** T-cell-mediated cytotoxicity assay in which target cancer cells (CaOV3, PA1 and MDA-MB-231) co-cultured with TCR/CD3 effector T cells were exposed to anti-EGFR/CD3 BiTE or DVD-Ig for 24h or 48h. After the incubation period, Promega CellTiterGlo reagent was added, and luminescence signals was measured using Promega GloMax plate reader. This experiment was performed in biological triplicates, and data is presented as mean ± SD. The differences between two groups were considered statistically significant when *p* < 0.05 (*p < 0.05; **p < 0.01). **(B)** Cancer cells (CaOV3 and MDA-MB-231) and T-cell co-culture were incubated with Cetuximab (Cet), DVD-Ig (DVD) and BiTE for 48h. WCL was collected and Western blotting was performed to detect the levels of cleaved caspase-3, -7. Actin was used as loading control. **(C)** Cancer cells (CaOV3 and MDA-MB-231) were treated with BiTE or DVD-Ig for 48h and then WCL was subjected to Western blotting to determine levels of cleaved caspase-3 and -7.

The capability of the anti-EGFR/CD3 BsAbs to induce T-cell-mediated killing of target cancer cells was assessed using a cell-based cytotoxicity assay. After co-culturing CaOV3, MDA-MB-231, and PA1 target tumor cells with T-cells, 50 nM BsAbs were added into the co-culture for 24h and 48h. The number of viable cells was determined by addition of CellTiterGlo reagent and measuring the luminescence. As shown in **Figure 4A**, both anti-EGFR/CD3 BsAbs formats induced statistically significant killing of CaOV3 cells at 24h incubation. However, only the BiTE format exhibited significant tumor cell killing of the PA1 and MDA-MB-231 cells. For the 48h incubation, both anti-EGFR/CD3 BsAbs formats induced statistically significant killing of all three target tumor cells. Additionally, comparative analysis of cytotoxicity data also revealed that BiTE formats induced significantly higher killing in CaOV3 and MDA-MB-231 cells than DVD-Ig format at 48h incubation. For the control, T cells were incubated with both anti-EGFR/CD3 BsAbs formats in absence of any target cells and the data showed no significant decrease in viability of T cells. Moreover, peripheral blood mononuclear cell (PBMC)–mediated killing of tumor cells by anti-EGFR/CD3 BsAbs were also assessed, and data showed that BiTE format induced more significantly killing of tumor cells compared with DVD-Ig format (**Supplementary Figure S3**). It is noted that PA1 cells express relatively low levels of EGFR as compared to the CaOV3 cells. Collectively, these data indicate that both anti-EGFR/CD3 BsAbs formats can induce significant cytotoxicity of target cancer cells, but the BiTE formats can trigger killing faster than DVD-Ig format.

### 3.5 Caspase-3 and -7 activation by anti-EGFR/CD3 BiTE format

To explore the T-cell-dependent cytotoxicity mechanism induced by anti-EGFR/CD3 BsAbs, we assessed the critical apoptotic markers, caspase-3 and -7 activities in T cell and target cancer cell co-culture system. Caspase-3, a crucial mediator of programmed cell death (apoptosis), catalyzes specific cleavage of cellular proteins and activates caspase-6, -7, and -9 to mediate apoptotic killing of cells (29, 30). As shown in **Figure 4B**, incubation of T cell and CaOV3 with BiTE and DVD-Ig increased the levels of cleaved caspase-3, and the levels of caspase-3 in cells exposure to the BiTE format was greater than the cells exposed to DVD-Ig. Interestingly, BiTE, but not DVD-Ig, mediated activation of cleaved caspase-3 in the MDA-MB-231 cells (**Figure 4B**). Similar to caspase-3, -7 also plays important role in execution phase of apoptosis. Incubation of co-culture systems with BiTE showed enhanced expression of cleaved caspase-7 fragments in both CaOV3 and MDA-MB-231 cells, but not for the DVD-Ig (**Figure 4B**). The anti-EGFR antibody cetuximab which was used as control in these experiments did not show any caspase activity in co-culture system. To confirm that T-cell engagement is required to elicit BiTE-induced caspase activity, caspase expression was examined in absence of T-cell culture (**Figure 4C**). Data from CaOV3 and MDA-MB-231 cells showed that BiTE or DVD-Ig treatment did not induce any caspase response in absence of T cells.

### 3.6 Granzyme B, perforin activity, and cytokine array

**Figure 5:**
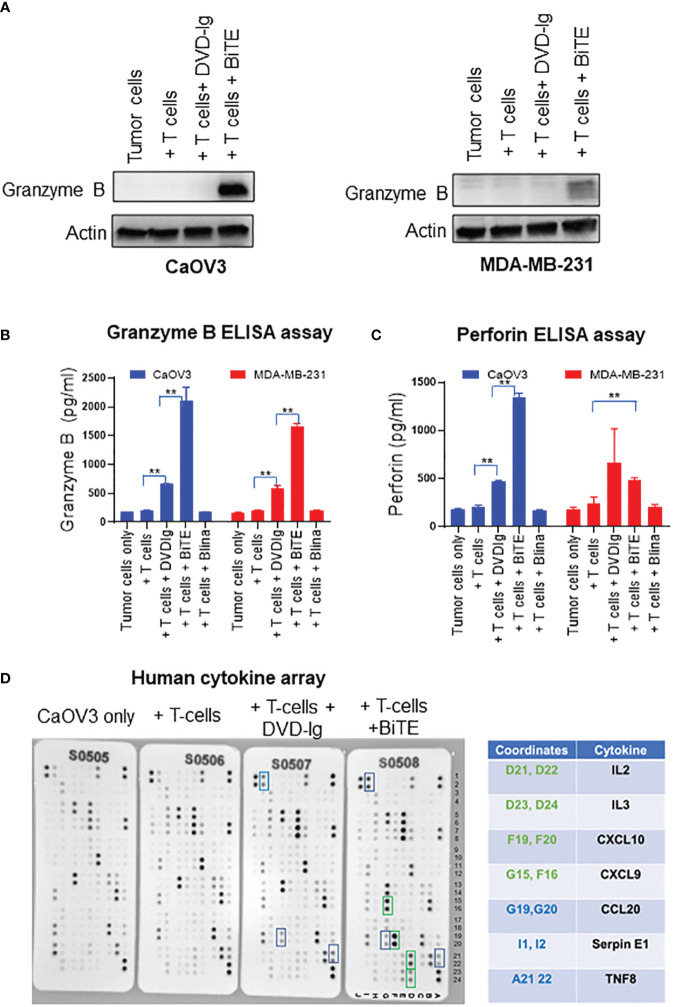
Granzyme/perforin activity and cytokine production by anti-EGFR/CD3 DVD-Ig and BiTE **(A)** Representative anti-granzyme B immunoblots using the WCL from co-culture of tumor cells (CaOV3 or MDA-MB-231) and T cells exposed to DVD-Ig or BiTE for 2 days. First lane represents tumors cell only. Actin was used as loading control. **(B, C)** Granzyme B and perforin measurement in co-culture supernatants was performed using ELISA kit. This experiment was performed in biological triplicates, and data is presented as mean ± SD. **p < 0.01 **(D)** Detection of human cytokines in co-culture supernatant of CaOV3 was performed using array kit. Green boxes (and associated green cytokine coordinates) represent increased dot intensity under BiTE treatment condition; blue boxes (and associated blue cytokine coordinates) represent increased array dot intensity under both DVD-Ig and BiTE treatment conditions.

Granzyme B is a caspase-like serine proteases that are stored in granules of T cells and NK cells, and released via secretory granule exocytosis with perforin, a pore-forming protein that induces immune-mediated cell death. Perforin facilitates movement of granzyme B into the cytoplasm of target cells by forming 5–20 nm pores, and after internalization, granzyme B activates the caspase-dependent mitochondrial apoptotic pathway that leads to the lysis of target cells (31). We assessed the granzyme B expression in WCL collected from co-cultured target cells and T cells exposed to that anti-EGFR/CD3 BsAbs. As shown in **Figure 5A**, the levels of granzyme B expression were drastically increased in BiTE-treated CaOV3 or MDA-MB-231 cells cocultured with T cells as compared to the cells exposed to the DVD-Ig- BsAbs format or control conditions. Media from the co-culture was subjected to ELISA to measure granzyme B and perforin production. As shown in **Figure 5B**, granzyme B production was significantly increased in both DVD-Ig and BiTE-treated CaOV3 and MDA-MB-231 cells co-cultured with T-cells. Comparatively, the BiTE format induced significant increase in levels of granzyme B production in CaOV3 and MDA-MB-231 cells co-cultured with T cells. Significant increase in perforin production was also found in CaOV3 + T cell treated with the BiTE formatted BsAb, but not treated with DVD-Ig formatted BsAb (**Figure 5C**). Moreover, cytokine array data (**Figure 5D**) obtained from supernatants of co-culture system showed that IL2, IL3, CXCL10, and CXCL9 were dramatically increased after the BiTE treatment, while a few cytokines (CCL20, SerpinE1 and TNF8) were slightly increased after both DVD-Ig and BiTE treatment. DVD-Ig failed to induce the release of IL2, IL3, CXCL10, and CXCL9 into cell culture media. Taken together, these findings suggest that although both T-cell-engaging bispecific antibodies retain their ability to induce granzyme B/perforin activity and enhance cytokine production, but the BiTE format induced more profound granzyme B/perforin activity and cytokine production as compared to DVD-Ig format.

### 3.7 Assessing thermal stability of anti-EGFR/CD3 BsAbs using HPLC and SDS-PAGE

**Figure 6:**
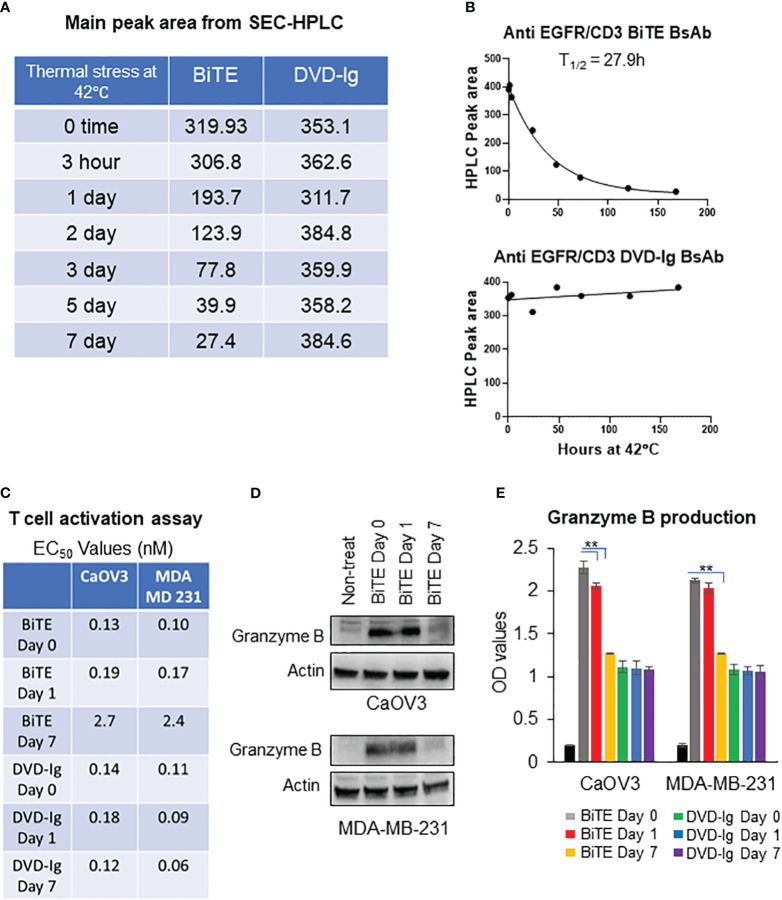
Thermal stability of anti-EGFR/CD3 BsAbs using HPLC and SDS-PAGE **(A)** Thermal-stressed BiTE and DVD-Ig were generated by placing protein samples at 42°C for the indicated timepoints and subjected to SEC-HPLC analysis to monitor for changes in thermal stability. The main peak area of BiTE and DVD-Ig format from SEC-HPLC analysis are presented in tabular format. **(B)** Main peak area from **(A)** are plotted against thermal stress timepoint using one-phase decay curve to determine half-life (T_1/2_). **(C)** Thermal-stressed BiTE and DVD-Ig samples at time days 0, 1, and 7 were subjected to T-cell activation assay using CaOV3 and MDA-MB-231 cells as described in **Figure 3**. EC_50_ values (nM) from T-cell activation assay are presented in tabular format for indicated samples. **(D)** Representative anti-granzyme B immunoblots using whole cell lysates from Co-culture of CaOV3 and T-cell exposed to Thermal-stressed BiTE samples (days 0, 1, and 7) for 48h. **(E)** Granzyme B production was measured in supernatants of co-culture system after treating with thermal stressed BiTE and DVD-Ig formatted BsAbs (days 0, 1, and 7). This experiment was performed in biological triplicates, and data are presented as mean ± SD. **p < 0.01.

To monitor the stability of the anti-EGFR/CD3 BsAb, the BsAbs were subjected to the thermal stress condition at 42°C for 0h, 1h, 3h, 24h, 48h, 72h, 120h, and 168h in storage buffer PBS at 7.4 using HPLC and SDS-PAGE analysis. After thermal stress, the samples were equally divided for analysis using HPLC and SDS-PAGE. As shown in the **Figure 6A**, **Supplementary Figure S2A**, HPLC main peak area for BiTE remained stable at the 3h timepoint followed by a decline that was observed from the 24h timepoint onwards. As time progressed, the HPLC main peak area for BiTE formatted BsAbs continued to decrease over time. On the other hand, DVD-Ig samples remain stable up to 168h (7 days), and no dramatic changes in HPLC main peak area for DVD-Ig samples were observed (**Figure 6A**, **Supplementary Figure S2A**). SDS-PAGE data (**Supplementary Figure S2B**) also revealed a similar stability profile for DVD-Ig and BiTE BsAbs formats as observed in HPLC assays. The HPLC main peak area data were plotted against the thermal stress incubation timepoints then, fitted using one-phase decay curve. These data indicated that BiTE BsAbs have approximate half-life of 27.9h under this stressed condition (42°C), whereas no significant degradation could be observed for DVD-Ig samples stored at stressed condition for 168h (**Figure 6B**). These data suggest that IgG backbone provides stability to molecule architecture in DVD-Ig format under thermal stress condition, whereas BiTE format shows rapid degradation profile due to lack of Fc portion.

### 3.8 Impact of thermal stress on anti-tumor potencies of BsAb

To determine the impact of thermal stress on the anti-tumor potency of the anti-EGFR/CD3 BsAbs, the BsAbs were stored at 42°C for 1 and 7 days then tested for the capability to activate T-cell activation by monitoring the expression levels granzyme B. As shown in **Figure 6C**, T-cell activation activity for the BsAbs with the BiTE format was dramatically reduced on day 7 as evidenced by ~20-fold increase in EC_50_ values compared to unstressed samples. In contrast the BiTE formatted BsAb loss in T-cell activation following thermal stress, the EC_50_ values were comparable between stressed and non-stressed DVD-Ig formatted BsAb, and the 7-day stressed DVD-Ig BsAb did not show any change in T-cell activation activity as compared to the non-stressed DVD-Ig BsAb (**Figure 6C**). Granzyme B activity was dramatically reduced in WCL of tumor cells (CaOV3 or MDA-MB-231 cells) and T-cell co-culture after 7-day treatment of BiTE under stressed condition (**Figure 6D**). Likewise, the production of granzyme B in co-culture supernatant was significantly reduced in 7-day thermal stressed BiTE formatted BsAb, whereas stressed DVD-Ig formatted BsAb didn’t show reduction in granzyme B production (**Figure 6E**). These data suggest that BiTE format is significantly more prone to thermal stress than DVD-Ig format due to the lack of Fc domain and anti-tumor potency is affected by thermal stress conditions in BiTE format but not in DVD-Ig format.

## 4 Discussion

The market approval of blinatumomab has led to an increased interest on T-cell-engaging bispecific antibodies. This group of bispecific antibodies is structurally complex and function as adaptors to transiently bridge T cells with tumor cells to mediate killing. Different formats of bispecific T-cell-engaging antibodies may have different intrinsic potencies not only from clinical effectiveness but also from safety and quality perspectives. The selection of the most appropriate formats of T-cell-engaging bispecific antibodies for clinical development is critical and affected by a sum of factors, including current understanding of quality attributes of different molecular formats, the biology of the targets, and the disease indications. Design of comprehensive and comparative studies are needed to gain a better understanding of how the differences in formats, especially with/without Fc portion, impact on functional characterization and bioassay development. This study focuses on structural and functional relationship of bispecific T-cell-engaging antibodies that target EGFR and CD3 in BiTE and DVD-Ig formats. Our results have revealed several interesting findings: (1) Higher binding activity toward CD3 on T cells does not increase T-cell-mediated killing of cancer cells or product potency, which is consistent with findings that higher binding activity on the T cells via CD3 is not proportional to their ability to induce the release of granzyme B, perforin and cytokines. (2) The Fc portion of bispecific T-cell-engaging antibody not only delays the action of T-cell-mediated killing but also reduces T-cell-mediated killing/potency, including the release of granzyme B, perforin and cytokines. It should be noted that the findings are obtained from cell-based assays. The Fc-containing bispecific T-cell engaging antibodies have longer serum half-life than Fc-less BiTE molecules in the *in-vivo* systems due to larger molecular size. Therefore, they may have different PK and PD profiles as compared to that of Fc less BiTE format. And another advantage for Fc containing bispecific T cell engaging antibody is that it may eliminate the need for continuous infusion. (3) The killing of cancer cells by anti-EGFR/CD3 BsAbs appears to be relatively independent of expression levels of target, more specifically, cancer cells with relatively lower levels of target expression, that is, EGFR, can also be killed by this type of bispecific antibodies. However, the conclusion is based only on this study and may not be appropriate to be generalized to other cell lines since T-cell killing activities of BsAbs targeting other tumor antigens could be different based on target structure and cell types. (4) The caspase-3 and -7 activation of caspase-mediated apoptotic pathway differentiates between the BiTE and DVD-Ig BsAb formats by unknown mechanism(s), suggesting that the format of T-cell engagers with or without Fc may need to be taken into consideration for clinical development whether caspase-3 and -7 mediated apoptotic pathway is involved in disease pathogenesis. (5) The T-cell activation assay shows that both formats have comparable T-cell activation potency in three tumor cell lines tested in this study and that the T-cell-dependent cytotoxicity assays demonstrated that the BiTE format exhibits significantly higher potency than that induced by DVD-Ig format. This suggests that T-cell activation assay may not be sensitive enough to differentiate differences in potency that reflects mechanism of action mediated by these two formats of T-cell-engaging bispecific antibodies. However, T-cell activation assay may be a stability indicating assay for BiTE format, but not DVD-Ig based on data obtained from thermostability (**Figure 6C**). (6) Thermostability of scFv fragments is an area of concern. Fragment-based antibody products are prone to low thermostability than conventional IgG based molecules and inefficient thermal stability can also significantly hamper the anti-tumor potencies of these products (32), consistent with our findings (**Figure 6**, **Supplementary Figure S2**). In this study, thermally stressed BiTE formatted BsAb also demonstrated significantly reduced T-cell activation assay and granzyme B activity in contrast to the DVD-Ig formatted BsAbs that exhibited a much better stability profile under stressed condition as compared to BiTE format and retains T-cell-mediated activity.

Thermal stabilization of antibody fragment is an important strategy to produce highly potent yet stable scFv-based therapeutic products, which can be achieved by several approached. One strategy is to introduce well-positioned disulfide bonds in scFv antibody scaffold that has been shown to stabilize single-domain antibodies to withstand extremely high melting temperature (33, 34). Other approaches are to produce bispecific fusion proteins in which BiTE-core molecules are fused with either Fc domain or human albumin to produce large size molecules that can also reduce kidney-mediated clearance. Several molecules with these strategies are currently in different stages of clinical development (35, 36).

In this study, we generated BiTE BsAb consisting of 6-histidine at C-terminal and utilized IMAC columns filled with pre-charged nickel ion resin to capture BiTE protein and then eluted with imidazole that can compete with his-tag binding to metal-charged resin. On the other hand, capturing of DVD-Ig BsAb protein was accomplished by a conventional IgG purification method using protein A affinity resin, which has enhanced binding capacity to the Fc region of IgG. The affinity tags, although very useful in protein purification strategies, may also cause issues with protein aggregation, misfolding, and immunogenicity induced by residual amino acids. It has also been suggested that manufacturability of antibody fragment-based products in CHO expression system may not be as efficient as conventional antibodies and production yield could be lower due to challenges associated with assembly, secretion, and stability (33, 37). In that regard, our investigation also revealed that BiTE based BsAb were produced at low titer range (mg/ml) as compared to DVD-Ig based BsAb using a transient expression system. This may suggest that the manufacture of scFv-based products is relativity much less efficient than IgG based products. It is worth to mention that we had initially tried to express BiTE from bacterium protein expression system; unfortunately, it did not work because BiTE format was completely insoluble.

Evaluating the CD3-based bispecific antibodies in *in-vivo* system has been very challenging because of the lack of suitable animal models. Due to the sequence identity between extracellular domains of human and murine CD3 is relatively low (58%), and human CD3 bispecific antibodies may not effectively activate mouse effector T cells to induce tumor cell killing (38, 39). Although immunodeficient xenograft murine models transfused with human immune cells were utilized in earlier studies, this model suffers several limitations including alloreactivity to xenografted tumor, donor-to-donor variation and difficulty in assessing the off-target toxicities (38). Recently, transgenic pre-clinical mouse models have been developed by replacing the murine CD3 components with human CD3 components (38). A recent *in-vivo* study using syngeneic mice model with humanized CD3 and target antigen CD20 demonstrated that the treatment with T-cell bispecific antibodies resulted in cytokine production profile and T cell activation reflecting the observations noted in human patients (39). These *in-vivo* findings are also in agreement with our investigation where we observed similar cytokine production profile and T-cell activation activity induced by anti-EGFR/CD3 BsAb.

Pre-clinical studies including our current investigation have consistently demonstrated an encouraging anti-tumor activity of T-cell engagers in solid tumors; however, clinical efficacy and durable responses in human patients remain limited due to the development of potential mechanisms of resistance (40). These resistance mechanisms include inadequate T-cell density in solid tumor microenvironment, and infiltrations of immunosuppressive cell populations and inhibitory cytokines. Additionally, upregulation of inhibitory immune-checkpoint molecules (such as PD-L1, CTLA-4, TIM3, LAG3, and TIGIT) can cause T-cell exhaustion and suppress the anti-tumor immune response. To overcome resistance and accomplish the successful clinical outcome, T-cell engagers may need to be combined with agents that block immune checkpoint upregulation, enhance T-cell proliferation activity and counterbalance immunosuppressive tumor environment (41). It would be interesting to further investigate if the structure of T-cell-engaging bispecific antibodies could possibly contribute to the resistant mechanisms that impact clinical efficacy.

Like bispecific T-cell-engaging antibodies, chimeric antigen receptor T-cell therapy (CAR-T therapy) relies on redirecting T-cell specificity against tumor antigen in MHC independent manner. In CAR-T therapy, patient’s peripheral blood T cells are first isolated and expanded and then genetically engineered to expressed CAR that recognizes tumor specific antigen (42). CAR-T treatment modalities suffer from several logistical limitations including need for leukapheresis, T-cell *ex-vivo* modifications and challenges with GMP manufacturability. Resistance mechanism to CAR-T therapy is similar to T-cell engagers that include lack of tumor antigen specificity, loss of tumor antigen, immunosuppressive tumor microenvironment, and upregulation of immune-checkpoint inhibitor (42). A combinatorial CAR-T and BiTE approach has been developed for EGFRvIII-expressing glioblastoma tumor in which CAR-T cells produced EGFR-targeting BiTE that can redirect CAR-T cells and mediate potent antitumor activity against heterogenous tumors, thereby overcoming tumor antigen loss (43). Further investigations are warranted to explore the possibilities of combining CAR-T therapy with T-cell engagers for better clinical outcome. The structural and functional relationship delineated from this study provides useful information for the design of bispecific T-cell-engaging antibodies for clinical and bioassay assay development.

## Data availability statement

The original contributions presented in the study are included in the article/**Supplementary Material**. Further inquiries can be directed to the corresponding authors.

## Author contributions

NM: Conceptualization, Data curation, Formal analysis, Funding acquisition, Investigation, Methodology, Validation, Visualization, Writing – original draft, Writing – review & editing. SA: Data curation, Formal analysis, Writing – review & editing. HP: Data curation, Formal analysis, Writing – review & editing. SD: Data curation, Formal analysis, Writing – review & editing. YS: Data curation, Formal analysis, Writing – review & editing. VF: Data curation, Formal analysis, Methodology, Writing – review & editing. TB: Data curation, Formal analysis, Methodology, Writing – review & editing. TJ: Data curation, Formal analysis, Methodology, Writing – review & editing. WW: Conceptualization, Data curation, Formal analysis, Funding acquisition, Investigation, Methodology, Project administration, Resources, Supervision, Validation, Visualization, Writing – original draft, Writing – review & editing.

## Supplementary material

Supplementary Figure 1 Histograms of flow cytometry binding experiments presented in **Figure 1E**. Line color coding for histograms: black = control; green = BiTE; red = DVD-Ig; blue = Cetux.

Supplementary Figure 2
**(A)** SEC-HPLC Chromatograms of thermal stressed BiTE and DVD-Ig samples as described in **Figure 6A**. **(B)** SDS-PAGE analysis of Thermal stressed BiTE and DVD-Ig as described in .

Supplementary Figure 3 PBMC-mediated killing of tumor cells induced by anti-EGFR/CD3 BsAbs. Target cancer cells (CaOV3, PA1, and MDA-MB-231) were co-cultured with PMBC at 10:1 ratio (PBMC/cancer cells) and then exposed to anti-EGFR/CD3 BiTE or DVD-Ig for 48h or 72h. Post incubation, Promega CellTiterGlo reagent was added, and luminescence signals were measured using Promega GloMax plate reader. This experiment was performed in biological triplicates, and data are presented as mean ± SD. The differences between two groups were considered statistically significant when *p* < 0.05 (**p* < 0.05; ***p* < 0.01).

Supplementary Figure 4 Unprocessed Western blot images of shown in **Figures 1F**, **2A**, and **2C**.

Supplementary Figure 5 Unprocessed Western blot images of shown in **Figures 4B, 4C**, **5A** and **6D**.
