# Supplementary figures and images for: Structural and functional characterization of IgG- and non-IgG-based T-cell-engaging bispecific antibodies
Source: Front Immunol. 2024 May 28;15:1376096. doi: 10.3389/fimmu.2024.1376096 (PMC11165055; doi:10.3389/fimmu.2024.1376096)

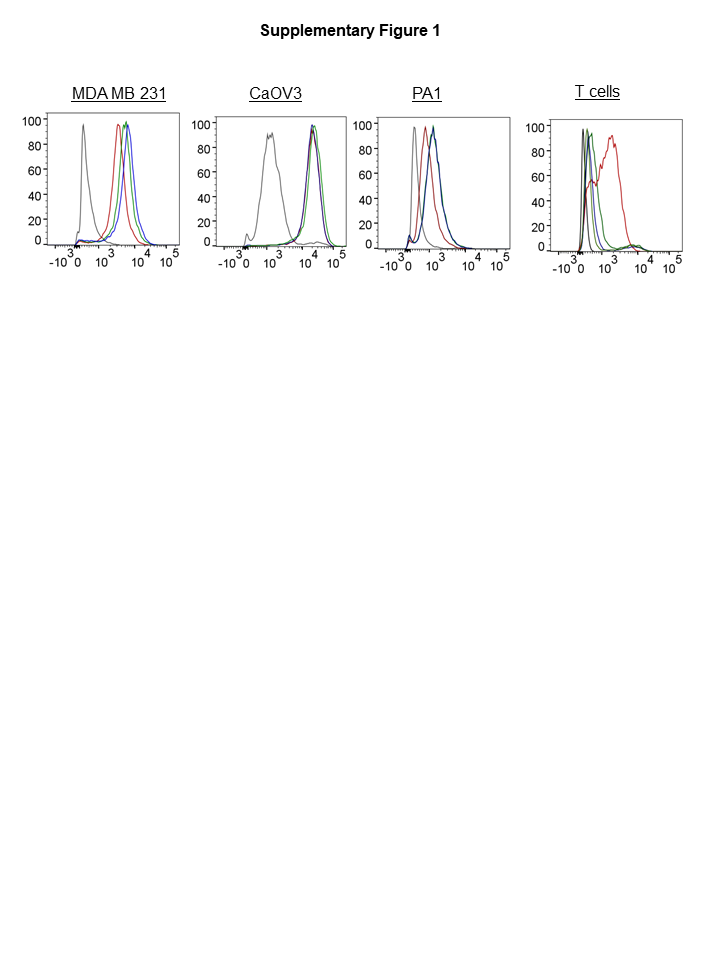

Supplement: Supplementary Figure 1 — Histograms of flow cytometry binding experiments presented in Figure 1E . Line color coding for histograms: black = control; green = BiTE; red = DVD-Ig; blue = Cetux. [file Image_1.tif]

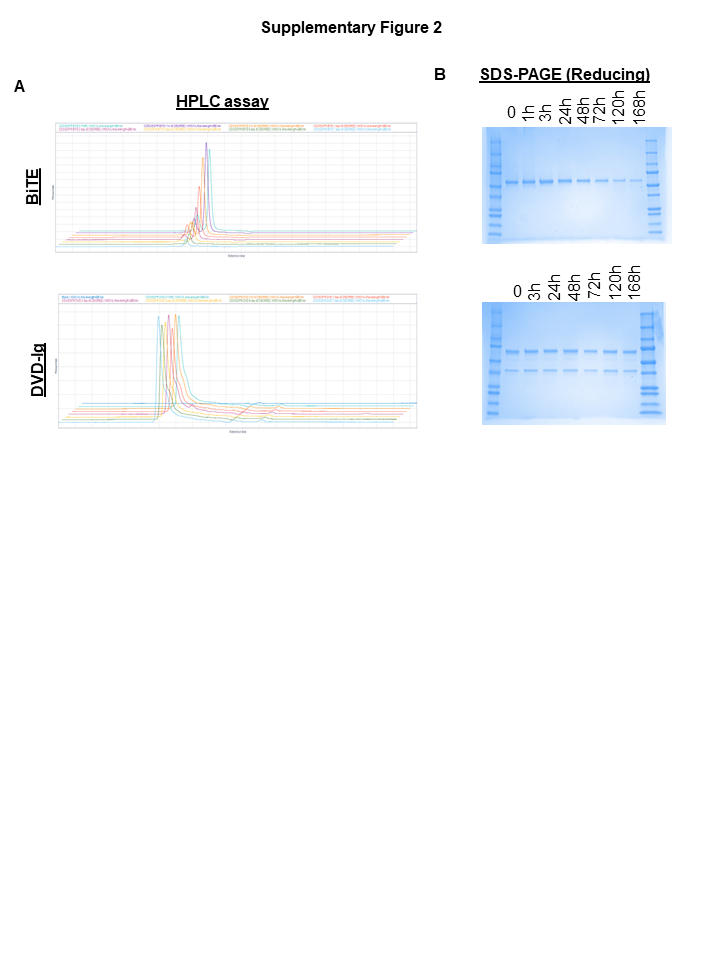

Supplement: Supplementary Figure 2 — (A) SEC-HPLC Chromatograms of thermal stressed BiTE and DVD-Ig samples as described in Figure 6A . (B) SDS-PAGE analysis of Thermal stressed BiTE and DVD-Ig as described in . [file Image_2.tif]

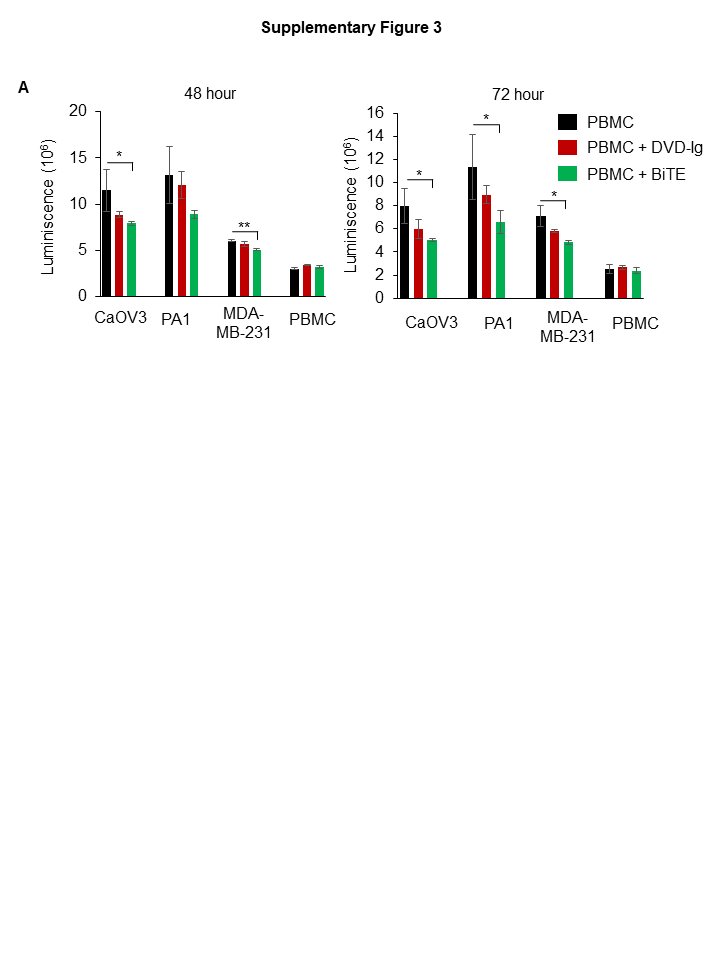

Supplement: Supplementary Figure 3 — PBMC-mediated killing of tumor cells induced by anti-EGFR/CD3 BsAbs. Target cancer cells (CaOV3, PA1, and MDA-MB-231) were co-cultured with PMBC at 10:1 ratio (PBMC/cancer cells) and then exposed to anti-EGFR/CD3 BiTE or DVD-Ig for 48h or 72h. Post incubation, Promega CellTiterGlo reagent was added, and luminescence signals were measured using Promega GloMax plate reader. This experiment was performed in biological triplicates, and data are presented as mean ± SD. The differences between two groups were considered statistically significant when p < 0.05 (*p < 0.05; **p < 0.01). [file Image_3.tif]

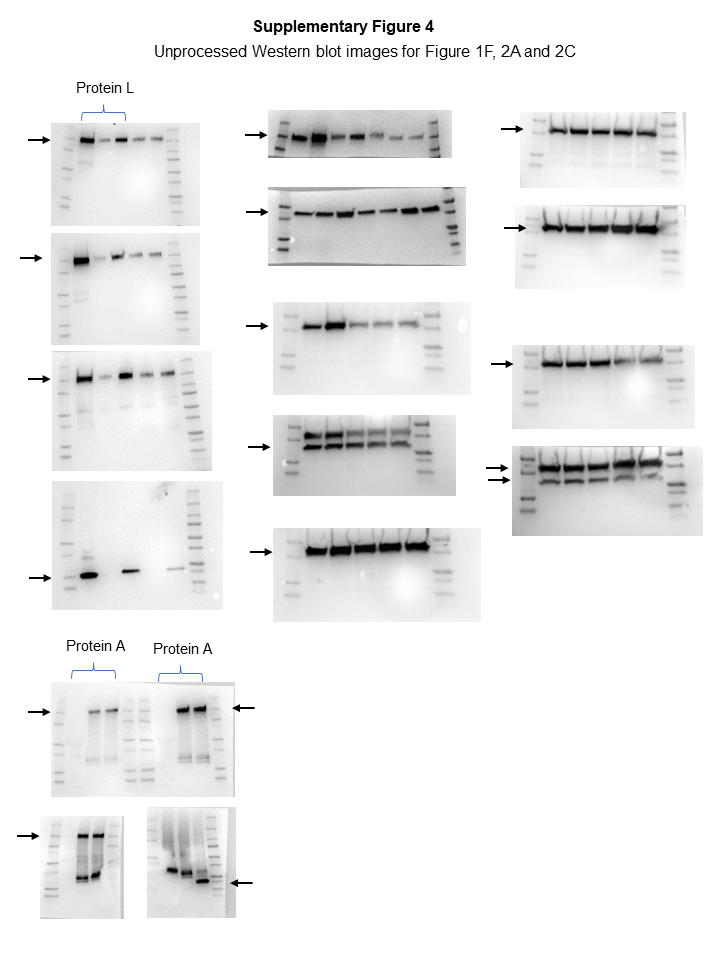

Supplement: Supplementary Figure 4 — Unprocessed Western blot images of shown in Figures 1F , 2A , and 2C . [file Image_4.tif]

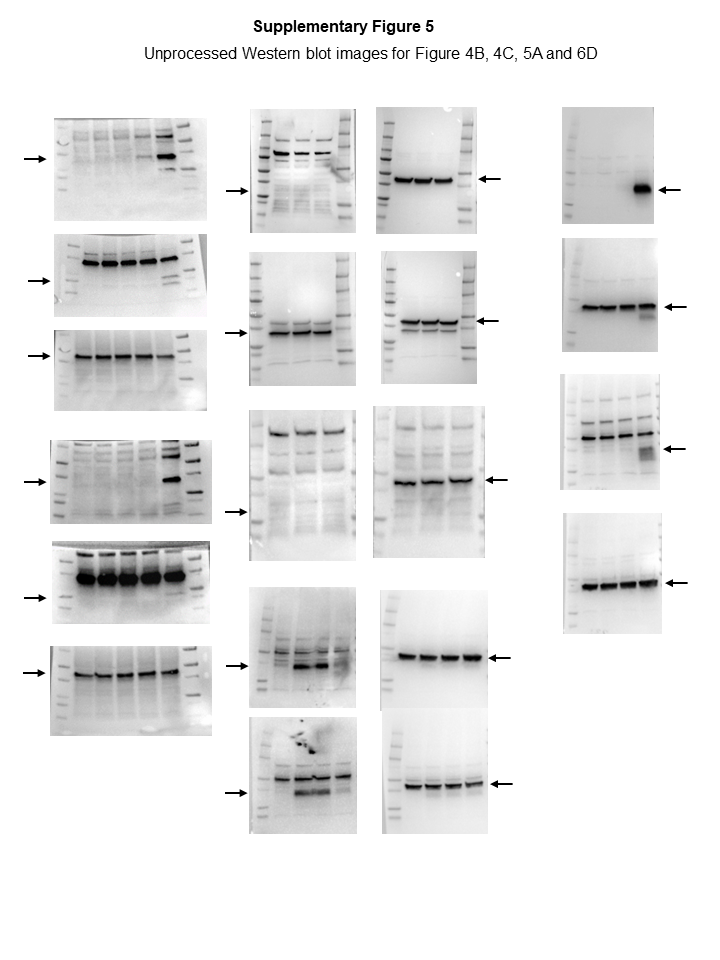

Supplement: Supplementary Figure 5 — Unprocessed Western blot images of shown in Figures 4B, 4C , 5A and 6D . [file Image_5.tif]
